# Synthetic Auxins Toxicity: Effects on Growth and Fatty Acid Composition in Etiolated and Green Spring Wheat Seedlings

**DOI:** 10.3390/molecules30214208

**Published:** 2025-10-28

**Authors:** Irina V. Lyubushkina, Kuzma A. Kirichenko, Marina S. Polyakova, Irina V. Polyanskaya, Natalya S. Zabanova, Anna V. Korsukova, Tamara P. Pobezhimova, Lyubov V. Dudareva, Evgenii G. Rikhvanov, Olga I. Grabelnych

**Affiliations:** 1 Siberian Institute of Plant Physiology and Biochemistry, Siberian Branch of the Russian Academy of Sciences, Irkutsk 664033, Russia; gargamelle@yandex.ru (K.A.K.); poljakova.m@gmail.com (M.S.P.); pavnatser@mail.ru (N.S.Z.); avkorsukova@gmail.com (A.V.K.); pobezhimova@sifibr.irk.ru (T.P.P.); laser@sifibr.irk.ru (L.V.D.); charmedwanderer@yandex.ru (E.G.R.); 2 Independent Researcher, Irkutsk 664023, Russia; irina.polyanskaya.01@mail.ru

**Keywords:** *Triticum aestivum* L., fatty acids, photosynthetic pigments, 1-naphthaleneacetic acid, 2,4-dichlorophenoxyacetic acid, clopyralid

## Abstract

Synthetic auxins are used in agriculture as herbicides worldwide, which leads to localized pollution and their potential entry into food crops during early developmental stages. *Triticum aestivum* L. is a major agricultural crop, and for this reason, understanding the mechanisms by which herbicides affect photosynthetic and lipid metabolic processes in wheat is crucial for assessing yield reduction risks. This study aimed to evaluate the toxic effects of three synthetic auxins, 1-naphthaleneacetic acid (NAA), 2,4-dichlorophenoxyacetic acid (2,4-D), and clopyralid (CLD) on growth parameters, membrane permeability, lipid peroxidation (LPO) product content, fatty acid (FA) profiles, and photosynthetic pigment levels in both etiolated and green spring wheat seedlings. FA content was assessed using gas chromatography-mass spectrometry. The results revealed that NAA and 2,4-D exerted the most pronounced inhibitory effects on seedling growth, whereas 2,4-D and CLD increased membrane permeability. In etiolated seedlings exposed to synthetic auxins, there was an elevation in FA content noted. Conversely, in green seedlings, exposure to all tested synthetic auxins led to a reduction in FA content, particularly affecting polyunsaturated fatty acids (PUFAs), as well as declines in chlorophyll and carotenoid levels. CLD reduced odd-chain fatty acid content (OCFAs) and very long-chain fatty acid content (VLCFAs) to undetectable levels. The increase in LPO products under the action of 2,4-D and CLD indicates oxidative stress as a possible cause of the decrease in PUFA content in green seedlings. These findings suggest that synthetic auxins have detrimental impacts on the photosynthetic apparatus of wheat, which in turn may have negative consequences for its productivity.

## 1. Introduction

**Figure 1:**
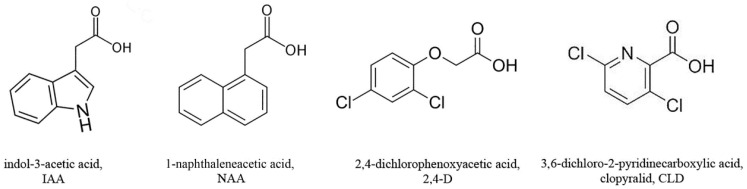
Natural auxin IAA (indol-3-acetic acid) and its synthetic analogues NAA (1-naphthaleneacetic acid), 2,4-D (2,4-dichlorophenoxyacetic acid), and CLD (3,6-dichloro-2-pyridinecarboxylic acid, clopyralid).

Auxins belong to a group of the most important plant growth-regulating hormones. They control almost all aspects of plant growth and development, encompassing root formation, stem elongation, and tropic growth among others [1,2]. In recent years, the important role of auxins as coordinators of hormonal crosstalk has been identified not only under normal conditions but also under stress influences [3]. The major natural auxin, indol-3-acetic acid (IAA), is a derivative of the amino acid tryptophan and is synthesized in plants with the participation of tryptophan aminotransferases and YUCCA (YUC) flavin-dependent monooxygenases (Figure 1) [2]. Synthetic analogues of natural auxins, namely, 1-naphthaleneacetic acid (NAA), 2,4-dichlorophenoxyacetic acid (2,4-D), and 2-methyl-4-chlorophenoxyacetic acid, were first developed in the 1940s (Figure 1) [4]. Clopyralid (3,6-dichloro-2-pyridinecarboxylic acid, CLD) was introduced by Dow AgroSciences LLC in 1975 (Figure 1) [5]. Over the past decades, these compounds have served as the basis for the agrochemicals used to prevent pre-harvest fruit drop in tree crops, obtain parthenocarpic fruits, and stimulate root formation. However, synthetic auxins have found their broadest application as herbicides for treating grain crops to control the growth of dicotyledonous weeds [6]. The utilization of 2,4-D and CLD in agriculture as systemic and selective herbicides stems from their ability to disrupt the hormonal networks [7]. These compounds act as agonists of natural auxin, mimicking its biological activities and inducing unregulated cellular proliferation, damage to vascular tissues, and deformation in roots and shoots [6]. NAA is used in agriculture to prevent fruit drop and improve fruit quality. It stimulates root formation and sugar accumulation [8,9]. There are studies indicating both lower toxicity of NAA [10] and similar toxic effects of NAA and 2,4-D on plants [11].

Despite being in use for more than 80 years, 2,4-D remains a component of approximately 1500 commercial agrochemicals [12]. Its enduring popularity stems from relatively high efficiency, selectivity, and low cost. However, prolonged global reliance on auxinic herbicides has led to the emergence of resistant weed species [13,14]. To counteract herbicide resistance, the range of auxin-based herbicides continues to expand. Manufacturers are increasingly incorporating adjuvants and additives that enhance the penetration of active substances, while also raising the concentration levels of active ingredients [15,16]. Such a strategy may have severe consequences for both ecosystem health and agricultural sustainability. Due to their relatively high resistance to synthetic auxins against physical and chemical environmental factors, the intensified use of synthetic auxins may cause localized pollution of soils and water [14]. Consequently, auxin-based herbicides can infiltrate cultivated plants, including cereals, during germination and seedling development, when these plants are most susceptible to such chemicals.

*Triticum aestivum* L. is one of the major agricultural crops and the most widely consumed cereal in the world [17]. The adaptability of wheat to environmental conditions has enabled its cultivation across a broad range of temperate climates [18]. Under optimal conditions, wheat yields can exceed 10 t/ha [17]. However, exposure to adverse environmental factors leads to substantial yield reductions [19]. Therefore, it is crucial to investigate all factors that may negatively affect wheat productivity, including the effects of herbicides. Although both 2,4-D and CLD have long been utilized in agricultural practices, research into their physiological impacts has predominantly centered around elucidating alterations in the metabolic pathways of dicotyledonous plants [7,20,21,22,23]. Conversely, information regarding the influence of synthetic auxins on the physiology and metabolism of cereals remains fragmented and contradictory [24,25,26,27]. However, studies have shown that 2,4-D at early developmental stages can cause stem shortening, suppress the development of leaves, roots, and spikelets, and reduce wheat yields [25]. Overlapping CLD treatments in winter wheat also resulted in visible plant damage and a 19% yield reduction [26]. Pyone et al. (2025) found no inhibitory effect of CLD on root and shoot growth in winter wheat [27]. Thus, exposure to synthetic auxins at early stages of plant growth may negatively affect the uniformity of cereal seedlings, impair fundamental metabolic processes, and ultimately compromise crop yields. Consequently, the specific effects of auxin-based herbicides on resistant plants during their early developmental stages require thorough and systematic investigation.

One pivotal aspect of cellular metabolism concerns lipid synthesis and membrane formation, as the membrane structure fundamentally determines cellular integrity, functionality, and homeostasis [28]. FAs serve as the predominant structural constituents of membrane lipids, substantially determining their structure, fluidity, and functional properties. FA composition and concentration vary during plant ontogeny and under different environmental factors [29,30,31]. Membrane structure and fluidity, which determine membrane permeability and the activity of membrane-bound enzymes, largely depend on PUFA content in membrane lipids [31]. High PUFA content, particularly linolenic acid (C_18:3_), is associated with increased plant tolerance to drought, salinity, and low and high temperatures [32,33,34,35,36]. PUFAs also serve as precursors of bioactive molecules, such as phytooxylipins and nitroalkenes, and participate in stress signaling [37,38,39,40]. Lipid and fatty acid composition can be altered by herbicides. Aryloxyphenoxypropionates and cyclohexanediones inhibit plastidic acetyl-CoA carboxylase; thiocarbamates and chloroacetanilides inhibit elongases and C_18:2_ desaturases; acetamides, chloroacetanilides, oxyacetamides, and tetrazolinone inhibit VLCFA synthesis [41,42,43,44,45]. Research on the effects of auxin-type herbicides on plant lipid metabolism is limited. NAA and 2,4-D enhanced lipid synthesis and accumulation in cells of the unicellular alga *Chlorella pyrenoidosa* under mixotrophic cultivation [46]. 2,4-D increased membrane permeability, LPO product levels, and reduced the C_18:2_/C_18:1_ fatty acid ratio in the fungus *Trichoderma harzianum*, which is widely used for protecting plants against pathogens and abiotic stresses [47]. No studies examining the effects of CLD on lipid synthesis in plants were found in the available literature.

This study investigates the effects of NAA, 2,4-D, and CLD on growth, membrane structure, and fatty acid composition in spring wheat (*Triticum aestivum* L.) seedlings. The experiments employed both etiolated and green seedlings to identify the effects of synthetic auxins, specifically on the formation of the photosynthetic apparatus. The use of three synthetic auxins with different toxicities—NAA, 2,4-D, and CLD—enabled differentiation of their individual effects and identification of shared characteristics typical of this group of chemicals.

## 2. Results

### 2.1. Synthetic Auxins Influence on Growth Characteristics of Spring Wheat Etiolated and Green Seedlings

**Figure 2:**
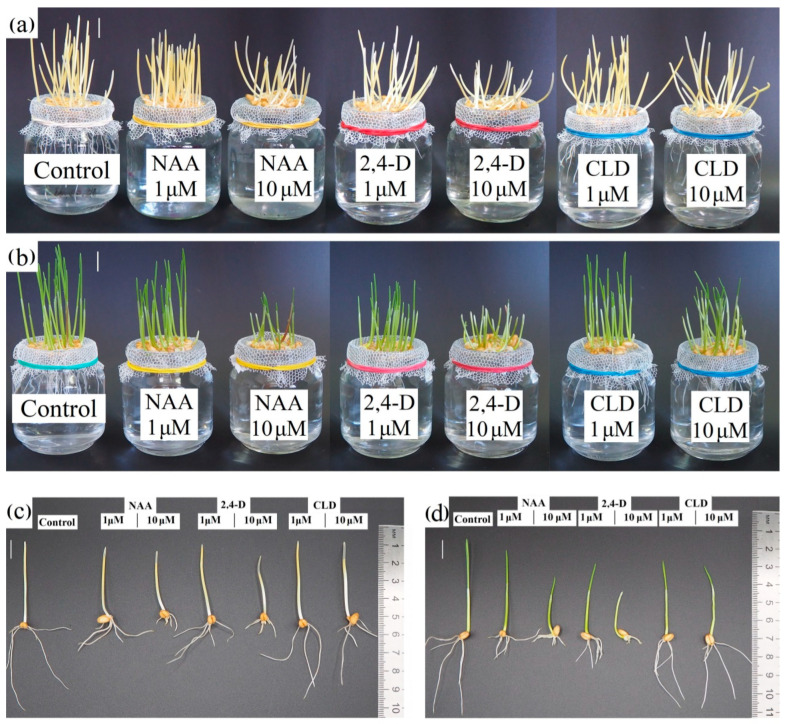
Photos of 4-day-old etiolated (**a**,**c**) and green (**b**,**d**) spring wheat seedlings: control and treated with synthetic auxins. Control—seedlings grown in water; NAA—seedlings grown in 1-naphthaleneacetic acid water solution; 2,4-D—seedlings grown in 2,4-dichlorophenoxyacetic acid water solution; CLD—seedlings grown in 3,6-dichloro-2-pyridinecarboxylic acid water solution. Concentration of synthetic auxin solutions: 1 μM and 10 μM. Scale bar is 10 mm.

**Figure 3:**
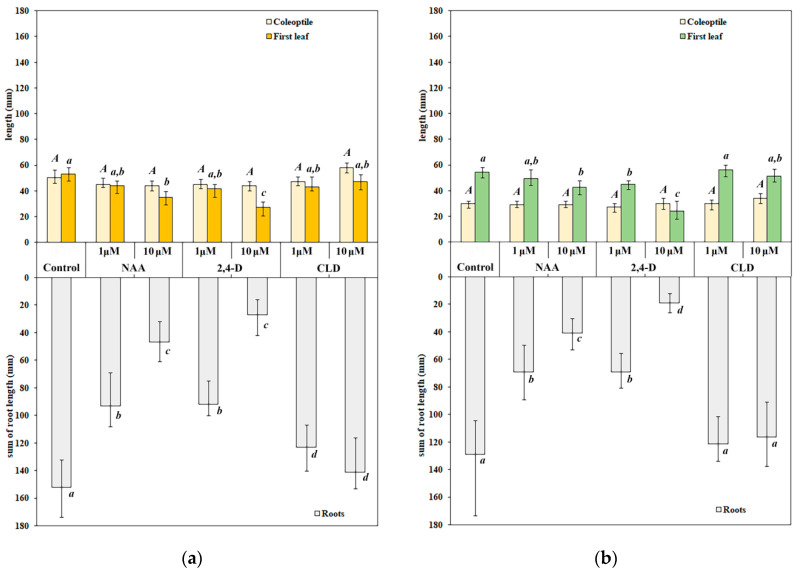
Changes in shoot and root length in 4-day-old etiolated (**a**) and green (**b**) spring wheat seedlings under synthetic auxin treatments. Treatment designations correspond to those in Figure 2. Data are presented as median (Q_50_) with interquartile range [Q_25_; Q_75_]. Distribution normality was assessed using the Shapiro–Wilk test. Statistical analysis employed the Kruskal–Wallis one-way ANOVA on ranks followed by Dunn’s multiple comparison test. Letters ‘a’, ‘b’, ‘c’ and ‘d’ indicate differences between variants. Uppercase letters indicate differences in coleoptile length, lowercase letters indicate differences in the lengths of the first leaf and roots. Statistically significant differences between treatments (*p* < 0.05) are indicated by different letters. *n* = 4.

The toxic effects of NAA, 2,4-D, and CLD were primarily assessed by measuring the inhibition of shoot elongation and total root length. The synthetic auxins had minimal impact on shoot elongation in etiolated seedlings. Neither NAA, 2,4-D, nor CLD affected coleoptile elongation at either concentration tested (Figure 2a,c; Figure 3a). Statistically significant inhibition of first leaf growth occurred only with 10 μM NAA and 10 μM 2,4-D treatments, reducing growth by 35% and 50%, respectively. CLD did not alter first leaf growth (Figure 2a,c; Figure 3a).

All tested auxins inhibited total root length growth in etiolated seedlings. Treatment with 1 μM NAA and 1 μM 2,4-D reduced root growth by approximately 40% (Figure 2a,c; Figure 3a). Both NAA and 2,4-D showed dose-dependent effects, with inhibition increasing to 70% and 80%, respectively, at higher concentrations. In contrast, CLD treatment produced concentration-independent inhibition of total root length growth, ranging from 10 to 20% regardless of the concentration used (Figure 2a,c; Figure 3a). Tested auxins did not affect the coleoptile elongation and only slightly inhibited first leaf growth, but the inhibition of total root length growth was more significant. The toxic effect of NAA and 2,4-D on etiolated seedlings was more pronounced than that of CLD.

The effects of synthetic auxins on the shoot elongation in green seedlings closely mirrored those observed in etiolated seedlings. NAA, 2,4-D, and CLD did not affect coleoptile elongation in green seedlings (Figure 2b,d; Figure 3b). First leaf elongation remained unchanged under 1 μM NAA treatment. However, 10 μM NAA produced a weaker inhibitory effect compared to etiolated seedlings, reducing first leaf elongation by approximately 20% (Figure 2b,d; Figure 3b). 2,4-D had a stronger inhibitory effect on seedlings grown in light than in darkness: it inhibited first leaf growth in green seedlings at 1 μM, with the effect intensifying at higher concentrations to approximately 20% and 55% inhibition at 1 μM and 10 μM, respectively (Figure 2b,d; Figure 3b). had no detectable effects on first leaf growth in either darkness or light.

The toxic effects of NAA and 2,4-D on total root length were enhanced in the light. In green seedlings, root growth inhibition by 1 μM NAA and 1 μM 2,4-D increased to nearly 50% (Figure 2b,d; Figure 3b). While 10 μM NAA reduced root length to the same extent as in etiolated seedlings (by 70%), the effect of 10 μM 2,4-D was slightly stronger in light, reaching 85% inhibition (Figure 2b,d; Figure 3b). CLD, on the contrary, had no visible effect on root growth under light conditions.

Thus, NAA and 2,4-D similarly affected shoot elongation and total root length in both etiolated and green seedlings, with dose-dependent responses and more pronounced growth inhibition in green seedlings. CLD showed no significant inhibitory effect on spring wheat seedling growth.

### 2.2. Synthetic Auxins Influence on Fresh and Dry Weight of Spring Wheat Etiolated and Green Seedlings

Auxin-induced changes in the accumulation of fresh and dry biomass of etiolated and green spring wheat seedlings correlated directly with changes in shoot and root growth rates. In etiolated seedlings, 10 μM NAA and 10 μM 2,4-D reduced fresh shoot biomass by 15% and 35%, respectively (Table 1). However, dry biomass accumulation was significantly reduced only by 10 μM 2,4-D treatment, with a decrease of 30%. Thus, the inhibition of shoot growth caused by NAA resulted primarily from suppressed cell elongation and acid growth. CLD had no effect on shoot biomass accumulation in etiolated seedlings.

Treatment with 1 μM and 10 μM NAA reduced fresh root biomass accumulation in etiolated seedlings by approximately 30% and 60%, respectively (Table 1). Dry root biomass accumulation was reduced only at a higher NAA concentration (10 μM), showing a 50% reduction compared to controls (Table 1). The herbicide 2,4-D was more potent, reducing fresh root biomass by 40% and 80%, and dry root biomass by 30% and 65%, at 1 μM and 10 μM, respectively (Table 1). Dry root biomass accumulation decreased by 30% and 65% under 1 μM and 10 μM 2,4-D treatment, respectively. Both NAA and 2,4-D demonstrated clear dose-dependent effects on root biomass accumulation. In contrast, CLD showed no effect on root biomass accumulation in etiolated seedlings.

In green seedlings, similar patterns were observed when studying the effects of synthetic auxins on shoot and root biomass growth, with NAA and 2,4-D exhibiting preserved dose-dependent responses, while CLD remained ineffective. Fresh shoot biomass accumulation was reduced by 15% at 1 μM NAA and 30% at 10 μM NAA (Table 1). Dry shoot biomass accumulation was reduced to only at 10 μM NAA, showing 25% inhibition. 2,4-D at 1 μM and 10 μM inhibited fresh shoot biomass by 20% and 45%, and dry shoot biomass by 10% and 35%, respectively (Table 1). Treatment with 1 μM and 10 μM NAA inhibited fresh root biomass accumulation by 30% and 65%, respectively (Table 1). The effects of NAA on dry root biomass accumulation were similar to those observed for dry shoot biomass: no changes were detected at 1 μM NAA, while 10 μM NAA reduced dry root biomass accumulation by 50%. 2,4-D at 1 μM and 10 μM inhibited fresh root biomass growth in green seedlings by approximately 30% and 70%, respectively (Table 1). At the same time, inhibition of dry root biomass accumulation under the action of 1 μM and 10 μM 2,4-D was 25 and 60%, respectively. CLD had no effect on biomass accumulation in shoots and a weak effect on fresh root biomass of green seedlings.

These findings demonstrate that NAA and 2,4-D inhibited both linear growth parameters and biomass accumulation in etiolated and green spring wheat seedlings, with 2,4-D exhibiting greater phytotoxicity. Treatment with 2,4-D reduced fresh and dry biomass growth. CLD showed no detectable effect on fresh or dry biomass accumulation in shoots and a weak effect on the fresh biomass of spring wheat seedlings’ roots. The toxicity of NAA and 2,4-D to green seedling growth and biomass accumulation may result from their impairment of photosynthetic apparatus formation.

### 2.3. Synthetic Auxins Influence on the Electrolyte Yield in Green and Etiolated Seedlings

**Figure 4:**
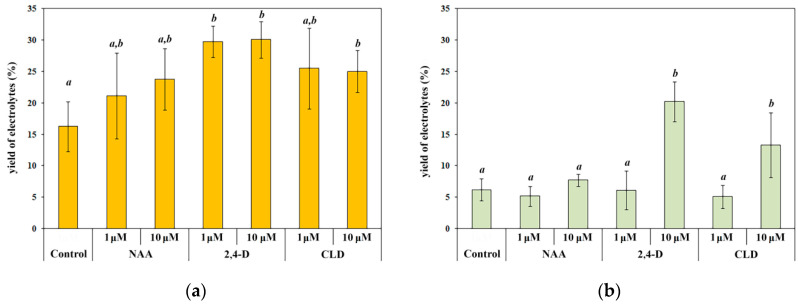
Yield of electrolytes in 4-day etiolated (**a**) and green (**b**) spring wheat seedlings under synthetic auxin treatment. Treatment designations correspond to those in Figure 2. Data are presented as the arithmetic mean (M) ± standard deviation (S.D.). Distribution normality was assessed using the Shapiro–Wilk test. One-way analysis of variance and the procedure of multiple comparisons of means according to the Fisher LSD method were used. Letters ‘a’ and ‘b’ indicate differences between variants. Statistically significant differences between treatments (*p* < 0.05) are indicated by different letters. *n* = 4.

The analysis of electrolyte yield allows for the assessment of changes in membrane permeability in response to external influence and provides an indirect measure of its severity. We found that in etiolated seedlings, 2,4-D and CLD increased electrolyte yield 2-fold and 1.5-fold, respectively (Figure 4a). The effect, however, was not dose-dependent. In green seedlings, an increase in electrolyte yield was exclusively induced by 10 μM 2,4-D and 10 μM CLD, showing 3-fold and 2-fold increases, respectively (Figure 4b). Treatment with 1 or 10 μM NAA did not increase electrolyte yield in either etiolated or green seedlings.

The results indicate that while CLD had no detectable effects on growth, it significantly altered cell membrane structure and permeability. NAA exerted a growth-inhibitory effect that was not associated with membrane alterations. In contrast, 2,4-D affected both growth parameters and membrane function.

### 2.4. Synthetic Auxins Influence on the Photosynthetic Pigments Content in Etiolated and Green Seedling Shoots

**Figure 5:**
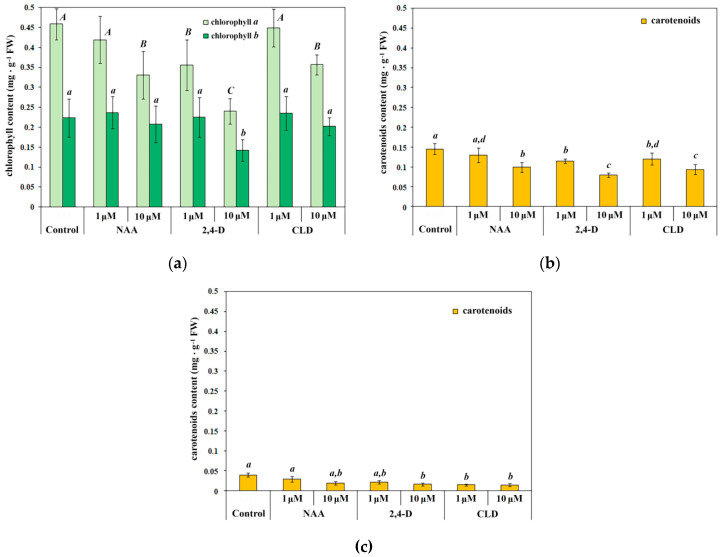
Content of chlorophylls *a* and *b* (**a**) and carotenoids (**b**,**c**) in 4-day-old spring wheat seedlings under synthetic auxin treatment. (**a**,**b**) Contents of chlorophylls and carotenoids in green seedlings; (**c**) content of carotenoids in etiolated seedlings. Treatment designations correspond to those in Figure 2. Data are presented as the arithmetic mean (M) ± standard deviation (S.D.). Distribution normality was assessed using the Shapiro–Wilk test. One-way analysis of variance and the procedure of multiple comparisons of means according to the Fisher LSD method were used. Letters ‘a’, ‘b’, ‘c’ and ‘d’ indicate differences between variants. Uppercase letters in (**a**) indicate differences in chlorophyll *a* content, lowercase letters in (**a**) indicate differences in chlorophyll *b* content. Statistically significant differences between treatments (*p* < 0.05) are indicated by different letters. *n* = 4.

To assess the toxic effects of synthetic auxins, the content of photosynthetic pigments in spring wheat green seedling shoots was determined after exposure to these auxins. All synthetic auxins examined reduced chlorophyll and carotenoid content in green seedlings, though with varying degrees of severity. Treatment with 1 μM NAA and 1 μM CLD did not alter chlorophyll a content, whereas 10 μM NAA and 10 μM CLD decreased chlorophyll a levels by approximately 30% (Figure 5a). The effect of 2,4-D was more pronounced: 1 μM 2,4-D reduced chlorophyll a content by 30%, while 10 μM 2,4-D treatment resulted in an almost 2-fold decrease (Figure 5a). Chlorophyll b content showed a different response pattern. Only 10 μM 2,4-D treatment significantly affected chlorophyll b levels, causing approximately 35% reduction. No changes in chlorophyll b content were observed following treatment with NAA or CLD at either concentration, or with 1 μM 2,4-D (Figure 5a).

Synthetic auxin treatment significantly altered carotenoid content in green seedlings. NAA exhibited concentration-dependent effects similar to those observed for chlorophyll: 1 μM NAA did not affect carotenoid levels, while 10 μM NAA caused approximately 30% reduction (Figure 5b). The inhibitory effect of 2,4-D and CLD was similar. Treatment with 1 μM 2,4-D and 1 μM CLD reduced carotenoid content by approximately 20%, while 10 μM concentrations of these compounds decreased carotenoid levels by 45% and 35%, respectively (Figure 5b).

In etiolated seedlings, the negative effect of synthetic auxins on carotenoid synthesis was also evident in the absence of light. Treatment with 10 μM NAA, as well as both concentrations of 2,4-D and CLD, reduced carotenoid content by approximately 45–65% (Figure 5c). Consistent with other experimental results, NAA exhibited the least pronounced toxic effects, influencing carotenoid synthesis in etiolated seedling shoots only at the higher concentration of 10 μM.

### 2.5. Synthetic Auxins Influence on the LPO Products Content in Etiolated and Green Seedling Shoots

**Figure 6:**
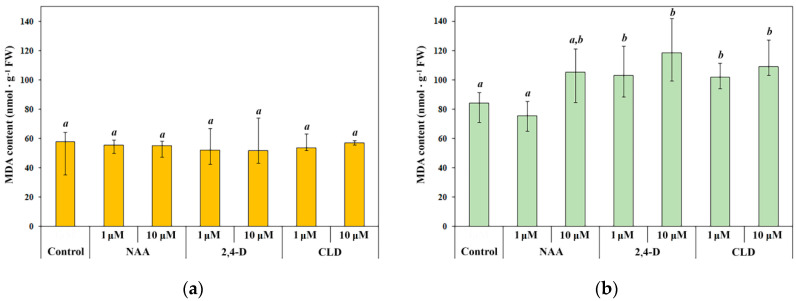
Content of malondialdehyde in 4-day etiolated (**a**) and green (**b**) spring wheat seedlings under synthetic auxin treatment. Treatment designations correspond to those in Figure 2. Data are presented as median (Q_50_) and interquartile range [Q_25_; Q_75_]. Distribution normality was assessed using the Shapiro–Wilk test. The Kruskal–Wallis one-way analysis of variance on ranks and all pairwise multiple comparison procedures (Dunn’s method) were used. Letters ‘a’ and ‘b’ indicate differences between variants. Statistically significant differences between treatments (*p* < 0.05) are indicated by different letters. *n* = 4.

The toxic effect of synthetic auxins could be associated with their influence on the redox metabolism of cells and the activation of LPO processes. It was shown that treatment of spring wheat seedlings with synthetic auxins did not affect LPO in the dark (Figure 6a). In green seedling shoots, LPO product content increased by approximately 20–25% under the action of 2,4-D and CLD in both studied concentrations. The effect of 2,4-D and CLD was not dose-dependent (Figure 6b). Consistent with other experimental parameters, NAA exhibited the least pronounced effect. Treatment with 1 μM NAA did not affect the intensity of LPO. The effect of 10 μM NAA did not lead to a statistically significant increase in the LPO products; however, the diagram shows a clear trend toward increased LPO activity. LPO product concentrations in green seedlings treated with 10 μM NAA were intermediate between control levels and the elevated concentrations observed in seedlings treated with 2,4-D and CLD (Figure 6b). Activation of LPO processes may explain the toxic effect of 2,4-D and CLD in green seedlings.

### 2.6. Synthetic Auxins Influence on Fatty Acid Content and Composition in Etiolated Seedlings

Analysis of etiolated spring wheat seedling shoots revealed fatty acids with chain lengths ranging from 14 to 22 carbon atoms (Table 2). The saturated fatty acids (SFAs) identified included myristic (C_14:0_), palmitic (C_16:0_), heptadecanoic (C_17:0_), stearic (C_18:0_), arachidic (C_20:0_), and behenic (C_22:0_) acids. Monounsaturated fatty acids (MUFAs) comprised hexadecanoic acid isomers (C_16:1_), oleic acid (C_18:1ω9_), *cis*-vaccenic acid (C_18:1ω7_), *cis*-13-octadecenoic acid (C_18:1ω5_), and gondoic acid (C_20:1ω9_) (Table 2). PUFAs were linoleic acid (C_18:2ω6_) and α-linolenic acid (C_18:3ω3_). The predominant SFAs in etiolated seedling shoots were palmitic (C_16:0_) and stearic (C_18:0_), while the key UFAs were oleic (C_18:1ω9_), linoleic (C_18:2ω6_), and α-linolenic (C_18:3ω3_). These five FAs constitute the main pathway of FA metabolism in plants and comprise the majority of both storage and membrane lipids. Therefore, we focused primarily on changes in these five FAs in response to synthetic auxin treatment.

We first examined changes in the five major FAs in etiolated seedlings following synthetic auxin treatment. Palmitic acid content increased 1.5-fold with 10 μM 2,4-D and 1 μM CLD, and nearly 2-fold with 10 μM CLD (Table 2). Stearic acid content increased 2-fold with 1 μM CLD and 3-fold with 10 μM CLD (Table 2).

Although 1 μM NAA did not affect UFA content, 10 μM NAA increased linoleic acid content 1.5-fold (Table 2). Similarly, 1 μM 2,4-D did not affect UFA content. The effect of 10 μM 2,4-D was more pronounced than that of 10 μM NAA. Treatment with 10 μM 2,4-D increased oleic and linoleic acid content 1.5-fold, and α-linolenic acid content 1.3-fold. Significant changes in major UFA content were also observed under the CLD action (Table 2). Treatment of etiolated seedlings with 1 μM and 10 μM CLD increased oleic acid content 1.3-fold and 1.7-fold, respectively. Additionally, 1 μM CLD increased linoleic and α-linolenic acid content approximately 1.5-fold (Table 2). However, 10 μM CLD did not affect the content of these fatty acids.

Of particular note are the changes in minor FA content in etiolated seedling shoots following synthetic auxin treatment. Treatment with 10 μM 2,4-D decreased cis-vaccenic acid (C_18:1ω7_) content 1.5-fold (Table 2). The effect of CLD on minor UFAs content depended on auxin concentration. Treatment with 1 μM CLD increased *cis*-vaccenic and *cis*-13-octadecenoic acid content nearly 2-fold. In contrast, 10 μM CLD did not affect *cis*-vaccenic acid content but decreased *cis*-13-octadecenoic acid content to trace levels (Table 2). Also noteworthy, palmitoleic acid and other hexadecenoic acid isomers decreased to trace levels following CLD treatment at both concentrations (Table 2). Of the SFAs in etiolated shoot seedlings, acids with an odd number of atoms (OCFAs) are particularly notable. Heptadecanoic acid was present in small quantities, while its precursor, pentadecanoic acid, was detected only in trace quantities. These FAs are of interest because their synthesis begins not with traditional acetyl-CoA, but with propionyl-CoA. Among all synthetic auxins studied, only CLD significantly affected the content of heptadecanoic acid: upon treatment with 10 μM CLD, its content decreased below the detection limit. Additionally, VLCFAs were detected in spring wheat shoots. These include SFAs—arachidic and behenic acids, as well as MUFA—gondoic acid. Their content remained unchanged after treatment with NAA and 2,4-D. However, treatment of etiolated seedlings with CLD at both concentrations tested decreased arachidic and behenic acid content to trace levels (Table 2). The content of gondoic acid dropped to trace values upon exposure to just 10 μM CLD. Meanwhile, when the seedlings were treated with 1 μM CLD, only a tendency towards a decrease in the content of this acid was observed.

Thus, synthetic auxins, notably 2,4-D and CLD, promoted de novo synthesis and desaturation of the major FAs. These observations are corroborated by marked increases in Σ_SFA_ and Σ_UFA_ (Table 2). Conversely, NAA exhibits weaker effects on FA synthesis: no detectable increase in Σ_SFA_ levels occurred upon NAA treatment, though a modest rise in Σ_UFA_ was observed after exposure to 10 μM NAA, suggesting increased activity in desaturation reactions, along with overall enhancement of FA synthesis and elongation. Desaturases utilize SFAs possessing corresponding carbon chain lengths as substrates. During the enzymatic reaction, these SFAs are actively consumed, thereby preventing their substantial accumulation, even under conditions of robust synthetic activity. The elongation and desaturation of fatty acids, serving as subsidiary processes in plant cell metabolism, were mostly inhibited by synthetic auxins, with this effect being particularly pronounced for CLD.

### 2.7. Synthetic Auxins Influence on Fatty Acid Content and Composition in Green Seedlings

Our study revealed that green shoots of spring wheat seedlings contain the same spectrum of fatty acids as etiolated shoots. Notwithstanding this similarity, the initial content of fatty acids, as well as the nature of auxin-induced changes in their profile, differed significantly compared to those observed in shoots from etiolated seedlings. The total content of FAs in green shoot seedlings was almost 2.5 times higher than that observed in the etiolated shoot seedlings (Table 2 and Table 3). This difference was mainly due to the higher level of UFAs. Σ_UFA_ in green seedlings were 2.7 times higher than in etiolated seedlings, while Σ_SFA_ in green seedlings exceeded that in etiolated ones only 1.5 times (Table 2 and Table 3). Almost all major FAs, including both SFAs and UFAs, namely palmitic, stearic, oleic, and linoleic acids, in green shoot seedlings were 2 times higher than in etiolated shoot seedlings (Table 2 and Table 3). The difference in α-linolenic acid content was even more significant, with C_18:3ω3_ concentrations 4-fold higher in green seedlings than in etiolated seedlings. Notably, several minor FAs exhibited higher concentrations in green seedlings compared to etiolated ones. Both behenic and *cis*-vaccenic acid levels increased approximately 3-fold in green seedlings compared to etiolated ones (Table 2 and Table 3).

Changes in FA content in green shoot spring wheat seedlings following treatment with synthetic auxins differed from those observed in etiolated seedlings treated with NAA, 2,4-D, and CLD. Unlike in etiolated seedlings, auxin treatment of green seedlings altered myristic acid levels. Treatment with 1 μM 2,4-D decreased C_14:0_ levels in green seedlings by nearly 2-fold, whereas treatment with 10 μM CLD increased levels by 1.6-fold (Table 3). These contrasting effects on a key precursor of all other fatty acids suggest that NAA, 2,4-D, and CLD differ in their mechanisms of action on fatty acid metabolism. The changes in FA content along the main metabolic FA pathway under the influence of synthetic auxins can be categorized into three distinct patterns. The first pattern was observed with 10 μM NAA treatment. In this treatment, all major FAs showed significant reductions, with the exception of stearic acid. Palmitic, oleic, and linoleic acid levels decreased by approximately 1.7–1.8 times; α-linolenic acid levels decreased by 2.1 times (Table 3). The second pattern, observed when green seedlings were treated with 1 μM 2,4-D and 1 μM CLD, showed that the content of α-linolenic acid decreased within a similar range as palmitic, oleic, and linoleic acids—approximately 1.6–1.8 times (Table 3). Similar changes in major FAs occurred under the action of 10 μM 2,4-D: palmitic, oleic, and α-linolenic acid levels decreased by 1.3–1.6 times, whereas linoleic acid content remained unchanged. The third pattern was observed after 10 μM CLD treatment. In this case, only α-linolenic acid was affected—its content decreased by 1.6 times, while other FAs in the main metabolic pathway remained unchanged (Table 3).

Treatment of green seedlings with synthetic auxins also negatively affected minor FA metabolism. Treatment with 10 μM CLD resulted in certain MUFAs being no longer detectable, including *cis*-13-octadecenoic acid (C_18:1ω5_) and hexadecenoic acid (C_16:1_) isomers (Table 3). Additionally, CLD treatment at both concentrations eliminated heptadecanoic acid, an odd-chain fatty acid. Changes in VLCFAs were particularly marked, specifically for behenic acid. The action of 1 μM NAA led to an almost 5-fold increase in behenic acid content in green seedlings, whereas 1 μM 2,4-D reduced levels of this acid by more than 18 times (Table 3). Notably, treatment with 1 μM NAA increased VLCFAs content while showing a trend toward decreased major FA content (Table 3). Although changes in major FAs were not statistically significant, this trend was sufficiently pronounced to suggest that NAA treatment shifts fatty acid synthesis and elongation pathways in green seedlings toward enhanced production of regulatory FAs, particularly behenic acid.

These results demonstrate that NAA, 2,4-D, and CLD had significantly different effects on FA synthesis, elongation, and desaturation in green spring wheat seedlings. Low doses of NAA promoted a shift in FA metabolism toward elongation processes and VLCFA formation. Higher NAA concentrations inhibited FA precursor synthesis, resulting in decreased levels of all major FAs. 2,4-D negatively affected both de novo FA synthesis and elongation/desaturation processes, indicated by decreased levels of both SFAs and UFAs. In contrast, CLD treatment primarily decreased UFA levels, possibly due to both inhibited desaturation processes and increased LPO.

Overall, analysis of FA content changes in etiolated and green spring wheat seedlings revealed that NAA, 2,4-D, and CLD increased the content of key SFAs and UFAs in etiolated seedlings but decreased their content in green seedlings. CLD had the most pronounced effect on OCFA and VLCFA content.

## 3. Discussion

Herbicide application remains fundamental to modern agriculture for controlling weed competition. Equally important is the degradation rate of these compounds, as herbicide persistence in soil during crop establishment can substantially impair yields. Over half of European soils now contain pesticide residues, representing a major environmental concern [48]. The half-life of compounds is determined primarily by their chemical structure, but is also influenced by environmental conditions such as temperature, humidity, and solar radiation exposure. For instance, 2,4-D is classified as a moderately persistent substance, with a half-life ranging from 20 to 312 days depending on environmental conditions [12]. In contrast, CLD exhibits a half-life of 2 to 14 months and demonstrates high resistance to both physical and chemical environmental factors, showing virtually no degradation under anaerobic composting conditions [49,50]. CLD contamination of soil and water resources represents a significant environmental challenge, as this compound resists metabolic degradation in plants and animals and can persist in feed or organic residues, even in countries where its use has been banned [51]. Furthermore, CLD exhibits high water solubility, readily migrates into groundwater systems, and can be transported over considerable distances, thereby posing potential risks to aquatic ecosystems [52,53]. Given the lack of comprehensive monitoring of pesticide residues—particularly auxin herbicides—investigating the effects of residual herbicides on crop growth and development represents a critical research priority. The synthetic auxin concentrations employed in this study (1 and 10 μM) are comparable to levels detected in natural water bodies and treated water supplies globally [54].

According to its chemical structure, the natural auxin IAA is a heterocyclic compound based on indole (Figure 1). Synthetic auxin analogues, including NAA and 2,4-D, mimic natural auxin activity but differ structurally: NAA is a non-heterocyclic compound derived from naphthalene, while 2,4-D is derived from phenoxyacetic acid (Figure 1). CLD is a picolinic acid derivative containing a pyridine heterocycle with a nitrogen atom in its structure (Figure 1). Despite these structural differences, NAA, 2,4-D, and CLD can mimic natural auxin action because they fulfill three essential molecular requirements for auxin bioactivity: a planar structure, a carboxyl group, and a specific distance between these features [55]. However, structural variations among synthetic auxins result in different degrees of toxicity and distinct metabolic effects in plants. Thus, although NAA is considered the archetypal synthetic auxin, several studies indicate its reduced genotoxic potential compared to 2,4-D and demonstrate its beneficial effects on plant physiological processes [8,9,10].

In general, plants respond to auxin herbicides similarly to high doses of natural auxins (e.g., IAA), but responses are more pronounced due to the slower degradation of synthetic auxins [56]. Natural auxin plays an important role in regulating plant architecture and organ development, including root system formation [57,58]. Local auxin maxima in the root apex maintain the quiescent center and stem cell pool [59]. Lateral root formation and development also occur under auxin control [60]. Therefore, exposure to auxin agonists through the root system during early developmental stages would be expected to significantly impact plant formation and growth, particularly by affecting root systems.

Consistent with this, our results showed that synthetic auxins exerted their strongest inhibitory effects on root growth, although the degree of inhibition varied significantly among the different auxins (Figure 2 and Figure 3). The most significant effects on both root and shoot growth were observed with 2,4-D and NAA, while CLD was weaker in this regard (Figure 2 and Figure 3). Primary manifestations of 2,4-D toxicity include thickening of roots and leaves, inhibition of their growth, and, in some cases, complete growth arrest, and structural and functional disruption of vascular tissues [61]. Notably, NAA exhibited an inhibitory effect comparable to that of 2,4-D in our experiments (Figure 2 and Figure 3), although some of the literature reports indicate lower toxicity for NAA [10]. Similar findings were reported by Da Costa et al., who observed that NAA suppressed adventitious root growth in *Arabidopsis thaliana* L., while 2,4-D not only inhibited growth but also promoted callus formation [62]. Consistent with our results, the effects of both NAA and 2,4-D on *A. thaliana* were dose-dependent [62].

The inhibitory effect of synthetic auxins, particularly NAA and 2,4-D, extended to shoot growth in our study (Figure 2 and Figure 3). Decreased linear growth rates were generally accompanied by reduced fresh biomass accumulation. Additionally, seedlings treated with 10 μM 2,4-D exhibited decreased dry biomass growth (Table 1). Notably, the inhibitory effects of NAA and 2,4-D on growth processes were more pronounced in green seedlings than in etiolated ones (Figure 2 and Figure 3, Table 1). The inhibitory effect of CLD on growth processes in spring wheat seedlings was rather weak compared to NAA and 2,4-D (Figure 2 and Figure 3, Table 1). The differences between the effects of NAA and 2,4-D versus CLD may be attributed to distinct metabolic pathways of these compounds within plant cells. One detoxification pathway for 2,4-D in cereals involves irreversible hydroxylation followed by conjugation with sugars [56].

Sugars serve not only as an energy source through ATP synthesis during their oxidation, but also as carbon skeletons for amino acid and protein synthesis. Their utilization in binding toxic compounds may contribute to the pronounced growth inhibition and reduced dry biomass accumulation observed in green wheat seedlings treated with 2,4-D (Figure 2 and Figure 3, Table 1). CLD is absorbed across membranes in its non-ionized form through passive diffusion and accumulates unchanged in the cytoplasm [63]. While CLD can accumulate in the cytoplasm via an ion-trapping mechanism, this binding is reversible and does not impede its translocation through the vascular system [63]. There is no evidence for CLD binding to water-soluble carbohydrates or amino acids.

Conversely, the greater physiological effects of NAA and 2,4-D result from their ability to mimic auxin signaling via the TIR1/AFB-Aux/IAA pathway [56,64]. This signaling pathway is initiated when auxin binds to the receptor proteins TIR1 (Transport Inhibitor Response 1) and AFB (Auxin Signaling F-Box) [65]. The conformation of the auxin binding site in TIR1 allows accommodation of synthetic auxin analogues, including NAA and 2,4-D, thereby modifying auxin signaling via the TIR1/AFB-Aux/IAA pathway [64]. Additionally, PIN-FORMED auxin transporters can transport several phenoxyacetic acid-based herbicides, including 2,4-D, across membranes using the same mechanism as endogenous auxins [66]. Such effects have not yet been detected for CLD.

The minor effects of CLD on wheat seedling growth suggest negligible biochemical and cellular transformations when exposed to this compound. Nonetheless, we have identified multiple CLD-induced changes in FA content (Table 2 and Table 3), which were not always accompanied by changes in membrane permeability (Figure 4). Specifically, the application of NAA caused marked changes in seedling growth and essential FAs levels, yet no changes in membrane permeability were observed (Table 2 and Table 3; Figure 2, Figure 3 and Figure 4). We propose that the increased efflux of electrolytes observed under the influence of 2,4-D and CLD arises not from biochemical modification of membrane lipid composition but instead results from direct interaction of these molecules with polar lipids. Indeed, the chemical structure of 2,4-D, particularly that of CLD, appears to facilitate such interactions. As previously reported by M. Flasiński and K. Hąc-Wydro, natural IAA binds more strongly to lipid monolayers than does NAA [67]. Since the naphthalene ring of the NAA molecule occupies a much larger volume than the indole system of IAA, its penetration between the polar headgroups of phospholipids becomes restricted [68]. At the same time, the phenolic ring of 2,4-D and the pyridine ring of CLD are much closer in volume to that of natural IAA, thereby allowing them to interact more easily with lipid molecules. In addition, the CLD molecule is capable of forming hydrogen bonds, which also facilitates its interaction with the polar headgroups of phospho- and glycolipid molecules [49].

De novo FA biosynthesis is a multistage complex process involving dozens of enzymes. Changes in FA synthesis occur during plant adaptation to environmental stressors. For instance, modifications in the expression of the gene encoding β-ketoacyl carrier protein reductase (KAR) affect membrane stability and, consequently, thermotolerance in rice [67]. Enhanced activity of FA desaturases, which catalyze the introduction of double bonds and the formation of UFAs, correlates with cold resistance in plants [69]. Treatment with synthetic auxins resulted in significant modifications to the individual fatty acid profile of spring wheat seedlings, thereby demonstrating the considerable efficacy of these compounds. The nature of the observed changes depended on the seedlings’ metabolic state (Table 2 and Table 3). In etiolated seedlings, synthetic auxin treatment resulted in enhanced FA synthesis, elongation, and desaturation, as evidenced by increased levels of both individual FA and total FA content (Table 2). A similar increase in PUFA content in heterotrophic tissues was observed in soybean zygotic embryos treated with exogenous auxins (NAA and 2,4-D) [70]. Of particular interest, NAA (10 mg/L) specifically increased the level of linoleic acid but not α-linolenic acid, a result that aligns with our findings (Table 2). The authors conclude that 2,4-D enhanced the efficiency of desaturation processes in extraplastidial compartments. Conversely, in green seedlings, a significant decrease in FA content was observed, primarily in PUFAs, specifically linoleic and α-linolenic acids (Table 3). Currently, no data indicate that 2,4-D or other auxin-type herbicides directly affect the activity of enzymes involved in fatty acid synthesis in plant green tissues. Instead, the reduction in specific FAs caused by these compounds is primarily attributed to oxidative stress [21,71].

In heterotrophic plant tissues, FA synthesis rates are substantially reduced, particularly for PUFAs. This occurs because the metabolic machinery that supplies precursors and cofactors is predominantly allocated to supporting the development of the chloroplast system [31]. Acetyl-CoA carboxylase, the initial enzyme of FA synthesis, is activated by light, and the rate of its activity determines the overall rate of lipid synthesis [72,73]. Consistent with this, the total FA content, especially USFA, was significantly lower in etiolated spring wheat seedlings compared to green ones (Table 2 and Table 3). In heterotrophic tissues, the FA synthesis machinery is localized mainly in proplastids, where NADH serves as the preferred cofactor over NADPH [74]. NADH is generated through the oxidation of seed storage compounds; consequently, no deficiency occurs during the initial stages of seedling development, and thus no limitation exists on the activity of the corresponding FA synthesis enzymes. Notably, in etiolated seedlings, treatment with 10 μM CLD resulted in the greatest increase in key SFAs (palmitic and stearic acids) and MUFA (oleic acid). Concurrently, VLCFA content decreased to undetectable levels (Table 2). These data suggest that CLD may inhibit VLCFA elongases, similar to acetamide, chloroacetamide, and oxyacetamide herbicides [42,43]. However, given the limited research on this mechanism, this hypothesis remains speculative and requires further investigation.

The fundamental distinction between etiolated and green wheat seedling metabolism lies in the absence of photosynthesis in the former; consequently, seed storage compounds represent the sole carbon source for synthesizing any organic substance. Current research has established that photosynthesis is the main carbon source for de novo FA synthesis in plants. For instance, pyruvate dehydrogenase in leaves provides the majority of acetyl-CoA required for FA synthesis [75]. In the present study, all synthetic auxins examined negatively affected photosynthetic apparatus development and function in spring wheat seedlings, as evidenced by decreased levels of chlorophylls, carotenoids, and α-linolenic acid (Figure 5, Table 3). The effects of NAA, 2,4-D, and CLD on carotenoid synthesis are quite remarkable, providing further evidence of structural and functional disruption to the photosynthetic apparatus. Carotenoids serve dual roles as light-harvesting pigments and as photoprotective stabilizers of thylakoid membranes [76,77]. Reduced carotenoid content negatively affects the formation of the photosynthetic apparatus, particularly light-harvesting complexes and photosystem reaction centers. Consequently, photosynthesis disruption caused by auxin agonists negatively affected FA synthesis, which further inhibited the formation and function of the photosynthetic apparatus. Interestingly, J. Lang et al. reported that CLD did not affect photosynthetic function in *Malva moschata*, likely because plants were treated at approximately 50 days of age, when vascular tissues and photosynthetic organs were already fully developed [78].

α-Linolenic acid within thylakoid membrane lipids enhances membrane fluidity and can become excited to facilitate electron transport under elevated membrane potential [79,80]. In contrast, free α-linolenic acid inhibits electron transport and photoreductive activity in both photosystems (PSs), with particularly pronounced effects on PS II [81]. Therefore, this fatty acid plays a crucial role in photosynthetic apparatus structure and function by actively regulating photosynthetic processes. Consequently, any change in α-linolenic acid levels affects overall plant cell metabolism. Various experimental models and plant species have demonstrated a link between PUFA content and photosynthesis. On one hand, high photosynthetic activity is a necessary factor for PUFA synthesis in green plant tissues. For example, when comparing two tea cultivars, Jincha 2 and Wuniuzao, higher levels of FA synthesis precursors, α-linolenic acid, and its derivatives (such as methyl dihydrojasmonate) were found in Jincha 2, which exhibited higher photosynthetic rates, stomatal conductance, and transpiration rates [82]. Conversely, high PUFA content in thylakoid membrane lipids makes them susceptible targets for ROS and contributes to photosynthesis disruption under stress conditions. This is illustrated by heat-tolerant rice cultivars, which exhibited reduced linoleic and α-linolenic acid content alongside lower electrolyte leakage, reduced malondialdehyde (MDA) accumulation, and maintenance of maximum PSII quantum yield (Fv/Fm) [83]. Similarly, the FA composition of thylakoid membrane lipids shifted toward higher proportions of SFAs and MUFAs in the brown macroalga *Undaria pinnatifida* following prolonged high-intensity light exposure [84]. Increased SFA content has also been observed in *Amaranthus caudatus* and wheat (*T. aestivum*) under drought conditions [85]. Thus, reduced PUFA content is not merely a consequence of impaired photosynthetic activity but also contributes to membrane stabilization by limiting lipid peroxidation intensity.

The decrease observed in the content of PUFAs, especially linoleic and α-linolenic acids, in green seedlings exposed to NAA, 2,4-D, and CLD may be attributed to several mechanisms: reduced synthesis of FA precursors (e.g., stearic acid C_18:0_), increased LPO, and decreased desaturase activity. Reduction in C18 PUFA precursor content was mainly observed with high-concentration NAA and 2,4-D treatments (Table 3). Increased LPO product accumulation occurred predominantly in seedlings treated with 2,4-D and CLD. Previous studies have shown that 2,4-D toxicity is largely determined by its ability to enhance ROS generation in cells [86,87]. Likely, this ROS accumulation under 2,4-D treatment results from increased activity of enzymes, including xanthine oxidoreductase, acyl-CoA oxidase, and lipoxygenase [72]. The latter two enzymes participate in USFA oxidation; therefore, their potential activation during treatment with 2,4-D likely contributed to the PUFA reduction we observed in green seedlings. These findings suggest there could be some mechanisms underlying PUFA reduction in treated seedlings. In green seedlings, NAA disrupted PUFA biosynthesis, probably by inhibiting the synthesis and elongation of SFAs, which serve as essential precursors for PUFA formation. In contrast, CLD reduced PUFA content through enhanced LPO. Probably, 2,4-D employed a dual mechanism, affecting PUFA biosynthesis and promoting LPO. If we assume that CLD reduces UFA content by activating LPO processes, it is noteworthy that in most cases this reduction was observed at 1 μM, but not at 10 μM CLD (Table 3). This differs significantly from the results obtained for NAA and 2,4-D, which more markedly reduced the UFA content in green seedling shoots at 10 μM (Table 3). Auxin-type herbicides are known to enhance the generation of ROS in plant tissues, which affects the activity of antioxidant defense enzymes in cells [86]. We suggest that low CLD concentrations were insufficient for the rapid activation of this defense system, but that an increased dose of CLD enhanced the activity of antioxidant enzymes. Our preliminary experimental data suggest this particular mechanism, but further verification is required.

While the direct effects of NAA, 2,4-D, and CLD on desaturase activity require further investigation, their disruption of the photosynthetic apparatus may indirectly affect soluble desaturase function. These enzymes, localized in plastid stroma, receive electrons from NADH via ferredoxin [88,89]. As ferredoxin reduction depends on non-cyclic electron transport through the thylakoid electron transport chain, any disruption of photosynthesis would be expected to compromise the activity of these soluble desaturases.

Additionally, synthetic auxins, especially CLD, significantly altered minor FA synthesis, including OCFAs and VLCFAs (Table 2 and Table 3). Given the crucial roles these specialized lipids play in membrane homeostasis, intercellular communication, cuticular wax biosynthesis, and seed germination [90], changes in their metabolism are likely to have profound implications for plant development.

## 4. Materials and Methods

### 4.1. Plant Material, Growth Conditions, and Synthetic Auxins Treatments

Etiolated and green 4-day-old seedlings of soft spring wheat (variety Novosibirskaya 29) were used. Seeds were rinsed and sterilized with 0.1% KMnO_4_ and moistened with water, and left to swell for 24 h. Then the seeds were rinsed once more and transferred to a mesh grid stretched over 100 mL vessels. The vessels contained either water (Control) or aqueous solution of 1 and 10 μM of the following synthetic auxins: 1-naphthaleneacetic acid (NAA), 2,4-dichlorophenoxyacetic acid (2,4-D), and 3,6-dichloro-2-pyridinecarboxylic acid (clopyralid, CLD) (Sigma-Aldrich, St. Louis, MO, USA) dissolved in water. These concentrations were selected based on preliminary experiments. The grids were positioned so that water or solution contacted the mesh by 1–2 mm. Germination proceeded for 4 days in growth chambers (Binder, Tuttlingen, Germany) under one of two conditions: a 16 h photoperiod (23 °C day/20 °C night) or continuous darkness (22–26 °C). The water and solutions were changed every 2 days. All physiological and biochemical parameters were analyzed using freshly collected samples. For the assessment of lipid peroxidation products and for lipid extraction, the samples were frozen in liquid nitrogen.

### 4.2. Determination of Growth Parameters

To assess the toxic effect of the synthetic auxins on spring wheat, growth was measured in both green and etiolated seedlings. After four days of germination, the lengths of the coleoptile, first leaf, and the total root system were measured. The degree of growth inhibition was calculated for each parameter (coleoptile, first leaf, and roots) using the following ratio: (Control Length − Treatment Length)/Control Length.

Fresh and dry shoot and root biomass were also measured. Fresh biomass was determined immediately after cutting shoots and roots. Dry biomass was measured after drying the samples in an oven at 72 °C for 48 h. Three independent experiments were conducted, with 50 seedlings per experiment.

### 4.3. Determination of Electrolyte Yield

For the determination of electrolyte yield, freshly cut seedling shoots (200 mg) were placed into 100 mL vessels containing distilled water (50 mL). The vessels were covered with foil to minimize evaporation and incubated for 24 h in darkness at room temperature. Evaluation of the electrolyte yield was assessed using the conductivity meter HI 8734 (Hanna Instruments Inc., Nusfalau, Jud. Salaj, Romania). The electrical conductivity was measured at room temperature and again after the samples were boiled for 20 min. Before each measurement, the volume of water in the vessel was readjusted to 50 mL. Electrolyte yield (V, %)was calculated using the formula V = 100 × (Lt/Lk), where Lt is the electrical conductivity after the 24 h incubation at room temperature, and Lk is the electrical conductivity of the same sample after boiling [91].

### 4.4. Determination of Photosynthetic Pigments Content

Chlorophyll and carotenoid contents in shoots (coleoptile + first leaf) were determined according to Gavrilenko, Zhigalova [92]. Freshly cut shoot samples (50 mg) were homogenized, and pigments were extracted with 1 mL of chilled 80% acetone containing MgCO_3_. The homogenate was transferred into the test tubes and left to extract for 24 h at 4 °C in darkness. Then the samples were centrifuged at 7000× *g* for 10 min at 4 °C. The extract was transferred to a clear tube; the residual pellet was mixed with a chilled 80% acetone (1 mL) repeatedly and again centrifuged. The procedure was repeated until the pellet became completely discolored. The final extract volume was 7 mL. If necessary, the volume was adjusted with the solvent.

Chlorophyll *a*, chlorophyll *b*, and carotenoid contents in the extract were determined spectrophotometrically at a wavelength of 665, 649, and 440 nm, respectively. Chlorophyll content was calculated using Werner’s formulas: for chlorophyll *a*—C_chl*a*_ = 11.63 × D665 − 2.39 × D649; for chlorophyll *b*—C_chl*b*_ = 20.11 × D649 − 5.18 × D665; for chlorophyll *a* + chlorophyll *b*—C_chl*a*+*b*_ = 6.45 × D665 − 17.72 × D649. Carotenoid content was calculated using Wettstein’s formula C_car_ = 4.695 × D440 − 0.268 × C_chl*a*+*b*_. Chlorophylls and carotenoid content were represented as C_car_, C_chl*a*_, C_chl*b*_, C_chl*a*+*b*_, mg × L^−1^. Pigment content (mg × g^−1^ FW) was calculated by the formula F = C × V/(P × 1000), where C is pigment concentration (mg × L^−1^), V is volume of extract (mL), and P is plant fresh weight (g).

### 4.5. Determination of Lipid Peroxidation Products

The content of LPO products in shoots was determined by measuring thiobarbituric acid-reactive products (TBA-RP), as described previously [93]. Freshly cut shoot samples (about 500 mg) were frozen in liquid nitrogen, homogenized in 2.0 mL of 0.1% TCA, and centrifuged at 12,000× *g* for 15 min. A 0.5 mL aliquot of the supernatant was combined with 1 mL of 0.5% (*w*/*v*) TBA in 20% TCA. The resulting mixture was incubated in a boiling water bath for 30 min. The reaction was terminated by rapid cooling on ice. Then, the samples were centrifuged at 12,000× *g* for 5 min (MiniSpin, Eppendorf, Hamburg, Germany). The absorbance of the resulting supernatant was measured at 532 and 600 nm (SmartSpec Plus, BioRad, United States). TBA-RP content was calculated using the extinction coefficient of TBA 155/(mM cm) after subtracting the nonspecific absorbance at 600 nm and expressed as nM × g^−1^ FW.

### 4.6. Lipid Extraction Procedure

Freshly cut shoots (~600 mg) were frozen in liquid nitrogen and ground. To extract lipids, 10–15 mL of chloroform: methanol mixture (1:2 *v*/*v*) containing ionol (2,6-di-tert-butyl-4-methylphenol, final concentration of 0.001%) was added to the samples [94]. For quantitative analysis of FA content, 10 μg of nonadecanoic acid (C_19:0_) was added to the samples as an internal standard. To enhance the separation of water-soluble components, 1–1.5 mL of 0.1% NaCl aqueous solution was added. The lower chloroform fraction containing common lipids was collected for analysis. Chloroform was removed under vacuum using an IR-1LT rotary evaporator (Labtex, Moscow, Russia). The dry lipid residue was re-dissolved in 2 mL of 5% H_2_SO_4_ in methanol for transesterification at 55–60 °C for 30 min. The reaction was then stopped by rapid cooling of the mixture. Hexane (4–5 mL) was added to separate the FA methyl esters [95]. The upper fraction of hexane containing FA methyl esters was evaporated to a volume of 200–400 μL. This concentrated sample was applied to aluminum silica gel plates (Sorbfil PTSH-AF-V, Krasnodar, Russia) for the purification of FA methyl esters in a chamber with benzene. To visualize the area containing FA methyl esters, a small portion of the plate was treated with 10% H_2_SO_4_ in methanol and heated. Purified FAs methyl esters were extracted from the appropriate area on the plate with chloroform and evaporated using an IR-1LT rotary evaporator (Labtex, Moscow, Russia) at 55 °C to obtain a dry residue. The resulting residue was dissolved in 500 μL of hexane for gas–liquid chromatography analysis.

### 4.7. Analysis of FA Methyl Esters

The sample of FA methyl esters in hexane (1 μL) was injected into a gas chromatography-mass spectrometer 5973N/6890N MSD/DS (Agilent Technologies, Santa Clara, CA, USA). The analysis employed a quadrupole mass detector with electron impact (EI) ionization at 70 eV. Detection was performed in a total ion current recording. Separation of individual ions of FA methyl esters was achieved using an HP-INNOWAX capillary column (30 m × 250 μm × 0.50 μm) (Agilent Technologies, Santa Clara, CA, USA) with polyethylene glycol as the stationary phase and helium as the carrier gas. The gas flow rate was maintained at 1 mL/min. Operating temperatures were as follows: evaporator, 250 °C; ion source, 230 °C; detector, 150 °C; and transfer line, 280 °C. Scan range was 41–450 amu. The flow separation was 5:1. Chromatographic separation was performed isocratically at 200 °C.

Individual FA methyl esters were identified by calculating the equivalent length of the aliphatic chain (ECL) and comparing mass spectra with the NIST 05 and Christie libraries. Identification was further confirmed by comparing retention times with those of authentic standards (Supelco^®^ 37 Component FAME Mix, Sigma-Aldrich, St. Louis, MO, USA). The relative content of FAs was determined by the internal normalization method and expressed as weight percent (wt%) of the total FA content in the sample, taking into account the FA response factors. Nonadecanoic acid (C_19:0_), which is absent in the sample, was used as the internal standard. The absolute content of each fatty acid (P_i_) was calculated from the total weight of FA methyl esters (FAME) in the sample (P _Total FAME_) and the relative percentage of that acid (C_i % rel_) using the following formula: P_i_ = P _Total FAME_*C_i wt%_/100. The content of FAs was expressed as μg × g^−1^ FW.

### 4.8. Statistical Analysis

At least four independent experiments were performed for each experimental variant. Statistical analysis was carried out using SigmaPlot v.14.0. software. The statistical significance of differences between variants was determined using ANOVA. Data are presented as arithmetic mean (M) ± standard deviation (S.D.) for normally distributed data or as median (Q_50_) with interquartile range [Q_25_; Q_75_] for non-normally distributed data. Normality of distribution was assessed using the Shapiro–Wilk test. For normally distributed data, one-way analysis of variance (ANOVA) was performed, followed by Fisher’s least significant difference (LSD) test for multiple comparisons. For non-normally distributed data, the Kruskal–Wallis one-way ANOVA on ranks was used, followed by Dunn’s test for pairwise multiple comparisons. Statistical significance was set at *p* < 0.05. In figures and tables, significant differences between variants are indicated by different letters.

## 5. Conclusions

This study demonstrates that CLD, unlike NAA and 2,4-D, did not significantly affect spring wheat seedling growth. However, both 2,4-D and CLD substantially increased cell membrane permeability, whereas NAA did not. The observed differences in toxic effects among NAA, 2,4-D, and CLD likely result from both the structural differences between the molecules and their distinct impacts on plant signaling and metabolic pathways. The stronger effects on membrane structure and activation of lipid peroxidation processes by 2,4-D and CLD likely account for their toxicity. The three synthetic auxins had distinct effects on FA metabolism that differed between etiolated and green spring wheat seedlings. In heterotrophic seedlings, NAA, 2,4-D, and CLD treatment enhanced FA synthesis, elongation, and desaturation processes. Conversely, in green seedlings, synthetic auxin treatment decreased both SFA and UFA content. A decrease in α-linolenic acid, chlorophylls, and carotenoids under synthetic auxin treatment indicates impaired photosynthetic apparatus formation and function, which would likely compromise wheat grain yield and quality. The decrease in PUFA content in NAA-treated green seedlings occurred mainly due to reduced synthesis and elongation of SFAs, which are precursors to PUFAs. CLD decreased PUFA content through activation of LPO processes, while 2,4-D affected PUFAs through both pathways. The results demonstrate the toxic effects of the synthetic auxins studied during early stages of spring wheat development and describe, for the first time, their effects on fatty acid metabolism and photosynthetic apparatus formation in this species. The data obtained in this study highlight the need for monitoring herbicide residues in soils and assessing their risks to the development and physiological status of major crops, including cereals. As the obtained results may be cultivar-specific, further research on various winter and spring wheat cultivars is required to identify general patterns.

## Figures and Tables

**Table 1:** Influence of synthetic auxin treatments on the fresh and dry biomasses of shoots and roots of 4-day-old etiolated and green spring wheat seedlings.

	Control	NAA, μM		2,4-D, μM		CLD, μM	
		1	10	1	10	1	10
Etiolated seedlings							
Fresh weight, mg							
**Shoot**	38.91 ± 3.04 ^a^	38.78 ± 3.58 ^a^	33.31 ± 6.46 ^b^	34.11 ± 1.96 ^a,b^	25.44 ± 2.59 ^c^	36.30 ± 2.83 ^a,b^	36.08 ± 3.01 ^a,b^
**Roots**	6.67 ± 1.60 ^a^	4.62 ± 0.51 ^b^	2.71 ± 0.82 ^c^	4.02 ± 0.31 ^b^	1.42 ± 0.24 ^c^	7.01 ± 1.44 ^a^	6.03 ± 1.36 ^a^
**Dry weight, mg**							
**Shoot**	4.10 ± 0.26 ^a^	3.99 ± 0.57 ^a^	3.49 ± 0.64 ^a^	3.64 ± 0.32 ^a^	2.79 ± 0.34 ^b^	3.77 ± 0.49 ^a^	3.68 ± 0.34 ^a^
**Roots**	0.50 ± 0.11 ^a^	0.42 ± 0.11 ^a^	0.25 ± 0.09 ^b,c^	0.34 ± 0.03 ^c^	0.17 ± 0.02 ^b^	0.60 ± 0.10 ^a^	0.53 ± 0.11 ^a^
**Green seedlings**							
**Fresh weight, mg**							
**Shoot**	30.73 ± 1.78 ^a^	26.79 ± 0.81 ^b^	21.96 ± 3.00 ^c^	23.99 ± 3.52 ^b,c^	17.32 ± 2.38 ^d^	28.33 ± 2.08 ^a^	29.57 ± 3.25 ^a^
**Roots**	5.89 ± 0.72 ^a^	4.02 ± 0.24 ^b^	2.08 ± 0.76 ^c^	3.99 ± 0.40 ^b^	1.81 ± 0.12 ^c^	5.58 ± 1.16 ^a,d^	4.67 ± 0.49 ^b,d^
**Dry weight, mg**							
**Shoot**	3.69 ± 0.39 ^a^	3.39 ± 0.17 ^a,b^	2.77 ± 0.41 ^b,c^	3.23 ± 0.18 ^b^	2.33 ± 0.35 ^c^	3.42 ± 0.14 ^a,b^	3.29 ± 0.26 ^a,b^
**Roots**	0.48 ± 0.09 ^a,c^	0.39 ± 0.12 ^a,c^	0.23 ± 0.05 ^b^	0.36 ± 0.03 ^c^	0.18 ± 0.02 ^b^	0.53 ± 0.14 ^a^	0.41 ± 0.04 ^a,c^

Data are presented as mean ± S.D. (n = 4). Fresh and dry biomasses contain the weight of 1 seedling. Letters ‘a’, ‘b’, ‘c’ indicate differences between variants. Values followed by different letters are statistically significantly different (*p* < 0.05). One-way ANOVA followed by Student-Newman-Keuls method. Control—seedlings grown in the water at 26 °C in the darkness; NAA—seedlings grown in solutions of 1-naphthaleneacetic acid; 2,4-D—seedlings grown in solutions of 2,4-dichlorophenoxyacetic acid; CLD—seedlings grown in solutions of 3,6-dichloro-2-pyridinecarboxylic acid, clopyralid.

**Table 2:** Comparative analysis of fatty acid content and composition in shoots of 4-day-old etiolated seedlings of spring wheat under exogenous auxin treatment.

Acid Formula	Control	NAA, μM		2,4-D, μM		CLD, μM	
		1	10	1	10	1	10
**C_14:0_**	2.24 ± 0.68	1.85 ± 1.20	2.65 ± 0.89	1.75 ± 0.86	2.34 ± 0.33	4.35 ± 2.60	6.42 ± 3.28
**C_16:0_**	**145.50 ± 34.27 ^a^**	**190.42 ± 17.12 ^a,b^**	**192.75 ± 37.24 ^a,b^**	**153.94 ± 23.88 ^a,b^**	**212.26 ± 13.72 ^b^**	**205.36 ± 48.93 ^b^**	**278.69 ± 72.72 ^c^**
**C_17:0_**	0.68 ± 0.12	0.90 ± 0.27	0.96 ± 0.32	1.14 ± 1.06	0.86 ± 0.28	-	-
**C_18:0_**	**27.52 ± 4.98 ^a^**	**45.81 ± 11.66 ^a,b^**	**35.63 ± 10.29 ^a^**	**33.72 ± 14.88 ^a^**	**34.46 ± 3.30 ^a^**	**59.16 ± 26.48 ^b^**	**76.51 ± 15.92 ^b^**
**C_20:0_**	0.84 ± 0.28	0.69 ± 0.06	1.01 ± 0.44	0.83 ± 0.21	0.90 ± 0.08	-	-
**C_22:0_**	6.59 ± 2.61	6.19 ± 1.96	8.33 ± 1.78	6.40 ± 0.43	12.82 ± 4.59	10.37 ± 4.55	15.49 ± 7.81
**C_16:1_^●^**	2.60 ± 1.21	2.24 ± 1.12	2.06 ± 0.30	1.58 ± 0.74	2.51 ± 0.95	-	-
**C_18:1ω9_**	**24.37 ± 5.54 ^a^**	**24.40 ± 5.99 ^a^**	**30.01 ± 5.56 ^a,b^**	**24.72 ± 3.72 ^a^**	**36.76 ± 2.30 ^b,c^**	**32.14 ± 7.63 ^b^**	**43.07 ± 8.17 ^c^**
**C_18:1ω7_**	5.83 ± 2.78 ^a^	5.42 ± 1.57 ^a^	7.16 ± 2.50 ^a^	4.40 ± 0.96 ^a^	2.06 ± 0.20 ^b^	13.00 ± 3.63 ^c^	7.31 ± 0.92 ^a^
**C_18:1ω5_**	2.45 ± 0.80 ^a^	3.16 ± 0.44 ^a^	3.18 ± 1.08 ^a^	2.24 ± 0.39 ^a^	3.29 ± 0.26 ^a^	4.99 ± 0.73 ^b^	-
**C_20:1ω9_**	2.24 ± 0.38	2.13 ± 0.04	2.30 ± 0.65	2.51 ± 0.73	2.68 ± 1.48	1.73 ± 0.29	-
**C_18:2ω6_**	**143.36 ± 20.81 ^a^**	**149.40 ± 18.74 ^a^**	**214.89 ± 37.33 ^b^**	**139.41 ± 28.08 ^a^**	**237.31 ± 34.81 ^b^**	**204.24 ± 35.08 ^b^**	**153.26 ± 24.80 ^a^**
**C_18:3ω3_**	**146.60 ± 27.00 ^a,b^**	**158.78 ± 19.92 ^a,b^**	**187.90 ± 39.80 ^a,c^**	**129.90 ± 3.27 ^a^**	**197.85 ± 32.61 ^b^**	**225.38 ± 48.84 ^b^**	**108.09 ± 20.35 ^a^**
**Σ_SFA_**	183.53 ± 38.47 ^a^	245.87 ± 24.26 ^a^	241.33 ± 47.26 ^a^	197.78 ± 36.87 ^a^	263.63 ± 18.41 ^b^	279.24 ± 69.05 ^b^	377.11 ± 93.65 ^c^
**Σ_UFA_**	327.45 ± 55.75 ^a^	345.52 ± 34.91 ^a^	447.50 ± 86.24 ^b^	304.76 ± 37.48 ^a^	482.46 ± 66.12 ^b^	481.48± 80.52 ^b^	311.73 ± 51.87 ^a^
**Σ_TFA_**	510.98 ± 96.25 ^a^	591.40 ± 58.29 ^a,b^	688.84 ± 130.27 ^b^	502.54 ± 68.07 ^a^	746.09 ± 84.53 ^b,c^	760.73 ± 108.59 ^c^	688.84 ± 114.01 ^b^

Designation. The content of fatty acids is represented as μg·g^−1^ of fresh weight. ^●^—palmitoleic acid and another isomer of hexadecanoic acid; Σ_SFA_—the sum of saturated fatty acids, Σ_UFA_—the sum of unsaturated fatty acids, Σ_TFA_—the sum of total fatty acids. Letters ‘a’, ‘b’ and ‘c’ indicate differences between variants. Different letters indicate statistically significant differences in fatty acid content between variants. Control—4-day seedlings grown in the water at 26 °C in the darkness; NAA—seedlings grown in solutions of 1-naphthaleneacetic acid; 2,4-D—seedlings grown in solutions of 2,4-dichlorophenoxyacetic acid; CLD—seedlings grown in solutions of 3,6-dichloro-2-pyridinecarboxylic acid, clopyralid. M ± S.D. *n* = 4–7.

**Table 3:** Comparative analysis of fatty acid content and composition in shoots of 4-day-old green seedlings of spring wheat under exogenous auxin treatment.

Acid formula	Control	NAA, μM		2,4-D, μM		CLD, μM	
		1	10	1	10	1	10
**C_14:0_**	2.93 ± 1.26 ^a,b^	2.93 ± 1.27 ^a,b^	2.22 ± 1.00 ^a,b^	1.41 ± 0.31 ^b^	3.37 ± 0.77 ^a^	2.80 ± 1.19 ^a,b^	4.78 ± 1.87 ^c^
**C_16:0_**	321.19 ± 83.17 ^a^*	**288.88 ± 77.44 ^a^**	**180.13 ± 32.99 ^b^**	**177.87 ± 12.80 ^b^**	**234.51 ± 30.36 ^b^**	**193.99 ± 13.97 ^b^**	**250.14 ± 55.16 ^a^**
**C_17:0_**	0.94 ± 0.55	1.84 ± 0.90	1.18 ± 0.37	0.92 ± 0.36	1.24 ± 0.13	-	-
**C_18:0_**	55.30 ± 9.87 *	**45.29 ± 21.03**	**29.84 ± 14.73**	**27.28 ± 3.46**	**29.10 ± 4.86**	**35.69 ± 9.43**	**48.82 ± 12.00**
**C_20:0_**	1.88 ± 0.47	3.66 ± 1.66	1.02 ± 0.10	1.15 ± 0.49	1.54 ± 0.72	1.28 ± 0.42	1.05 ± 0.28
**C_22:0_**	21.06 ± 7.32 ^a^*	114.71 ± 27.42 ^b^	12.53 ± 2.51 ^a^	1.14 ± 0.40 ^c^	18.06 ± 2.65 ^a^	16.77 ± 4.27 ^a^	27.28 ± 12.69 ^a^
**C_16:1_^●^**	5.94 ± 3.12	7.86 ± 3.17	3.77 ± 1.02	4.66 ± 1.32	6.00 ± 2.95	3.60 ± 2.03	-
**C_18:1ω9_**	49.75 ± 15.16 ^a^*	**46.23 ± 10.96 ^a^**	**30.38 ± 2.97 ^b^**	**30.82 ± 1.17 ^b^**	**38.65 ± 9.03 ^a,c^**	**31.98 ± 2.23 ^b,c^**	**45.59 ± 1.19 ^a^**
**C_18:1ω7_**	16.11 ± 7.98 *	10.97 ± 3.85	7.95 ± 4.30	6.51 ± 1.83	9.75 ± 4.44	10.96 ± 6.21	9.42 ± 4.95
**C_18:1ω5_**	7.59 ± 5.63	4.11 ± 0.44	3.46 ± 1.32	3.16 ± 1.31	5.64 ± 1.07	4.23 ± 0.99	-
**C_20:1ω9_**	3.67 ± 1.88	5.61 ± 1.18	2.77 ± 0.82	2.87 ± 0.08	4.01 ± 1.53	3.13 ± 0.65	3.22 ± 0.92
**C_18:2ω6_**	283.90 ± 56.47 ^a^*	**248.18 ± 51.44 ^a^**	**171.88 ± 33.60 ^b^**	**172.55 ± 15.13 ^b^**	**278.14 ± 59.36 ^a^**	**175.14 ± 25.85 ^b^**	**224.77 ± 59.24 ^a^**
**C_18:3ω3_**	550.16 ± 109.97 ^a^*	**469.22 ± 141.28 ^a^**	**263.10 ± 69.64 ^b^**	**303.99 ± 50.79 ^b^**	**329.15 ± 76.17 ^b^**	**313.95 ± 74.23 ^b^**	**304.65 ± 115.63 ^b^**
**Σ_SFA_**	399.34 ± 100.68 ^a,b^	456.77 ± 110.27 ^b^	226.92 ± 45.75 ^c^	209.79 ± 16.34 ^c^	293.38 ± 40.65 ^c,d^	249.90 ± 14.65 ^c^	331.56 ± 59.07 ^a,d^
**Σ_UFA_**	896.03 ± 188.77 ^a^*	793.65 ± 200.55 ^a^	484.92 ± 104.39 ^b^	525.64 ± 64.74 ^b^	674.13 ± 146.43 ^b^	558.46 ± 95.39 ^b^	611.87 ± 149.23 ^b^
**Σ_TFA_**	1293.89 ± 274.15 ^a^*	1224.83 ± 303.01 ^a^	710.35 ± 119.40 ^b^	734.73 ± 49.96 ^b,c^	964.47 ± 164.31 ^c^	793.59 ± 96.10 ^b,c^	919.06 ± 169.24 ^b,c^

Designation. The content of fatty acids is represented as μg·g^−1^ of fresh weight. ^●^—palmitoleic acid and another isomer of hexadecanoic acid; Σ_SFA_—the sum of saturated fatty acids, Σ_USFA_—the sum of unsaturated fatty acids, Σ_TFA_—the sum of total fatty acids. Letters ‘a’, ‘b’ and ‘c’ indicate differences between variants. Different letters indicate statistically significant differences in fatty acid content between variants. ‘*’ indicate statistically significant difference between etiolated (Table 2) and green (Table 3) control seedlings. Control—4-day seedlings grown in the water at 26 °C in the darkness; NAA—seedlings grown in solutions of 1-naphthaleneacetic acid; 2,4-D—seedlings grown in solutions of 2,4-dichlorophenoxyacetic acid; CLD—seedlings grown in solutions of 3,6-dichloro-2-pyridinecarboxylic acid, clopyralid. M ± S.D. *n* = 4–7.

## Data Availability

The original contributions presented in the study are included in this article. Further inquiries can be directed to the corresponding author.

## Abbreviations

The following abbreviations are used in this manuscript:

CLD	3,6-Dichloro-2-pyridinecarboxylic acid, clopyralid
2,4-D	2,4-Dichlorophenoxyacetic acid
FAs	Fatty acids
IAA	Indol-3-acetic acid
LPO	Lipid peroxydation
MUFAs	Monounsaturated fatty acids
NAA	α-Naphthylacetic acid
OCFAs	Fatty acids with odd carbon numbers
PUFAs	Polyunsaturated fatty acids
SFAs	Saturated fatty acids
VLCFAs	Very long-chain fatty acids
UFAs	Unsaturated fatty acids
